# PARP3 is a sensor of nicked nucleosomes and monoribosylates histone H2B^Glu2^

**DOI:** 10.1038/ncomms12404

**Published:** 2016-08-17

**Authors:** Gabrielle J. Grundy, Luis M. Polo, Zhihong Zeng, Stuart L. Rulten, Nicolas C. Hoch, Pathompong Paomephan, Yingqi Xu, Steve M. Sweet, Alan W. Thorne, Antony W. Oliver, Steve J. Matthews, Laurence H. Pearl, Keith W. Caldecott

**Affiliations:** 1 Genome Damage and Stability Centre, School of Life Sciences, University of Sussex, Science Park Road, Falmer, Brighton BN1 9RQ, UK; 2 Cancer Research UK DNA Repair Enzymes Group, Genome Damage and Stability Centre, School of Life Sciences, University of Sussex, Science Park Road, Falmer, Brighton BN1 9RQ, UK; 3 CAPES Foundation, Ministry of Education of Brazil, Brasilia/DF 70040-020, Brazil; 4 Cross-faculty NMR centre, Department of Life Sciences, Faculty of Natural Sciences, Imperial College London, London SW7 2AZ, UK; 5 Institute of Biomedical and Biomolecular Sciences, University of Portsmouth, St Michael's Building, Portsmouth PO1 2DT, UK

## Abstract

PARP3 is a member of the ADP-ribosyl transferase superfamily that we show accelerates the repair of chromosomal DNA single-strand breaks in avian DT40 cells. Two-dimensional nuclear magnetic resonance experiments reveal that PARP3 employs a conserved DNA-binding interface to detect and stably bind DNA breaks and to accumulate at sites of chromosome damage. PARP3 preferentially binds to and is activated by mononucleosomes containing nicked DNA and which target PARP3 trans-ribosylation activity to a single-histone substrate. Although nicks in naked DNA stimulate PARP3 autoribosylation, nicks in mononucleosomes promote the trans-ribosylation of histone H2B specifically at Glu2. These data identify PARP3 as a molecular sensor of nicked nucleosomes and demonstrate, for the first time, the ribosylation of chromatin at a site-specific DNA single-strand break.

## Abstract (web summary)

Chromosomal single-strand DNA breaks occur frequently and require repair to avoid disease outcomes. Here, the authors show that in bird cells, PARP3 accelerates this repair, and use structural biology and cell biology techniques to reveal details of the mechanism of action.

## Graphical abstract

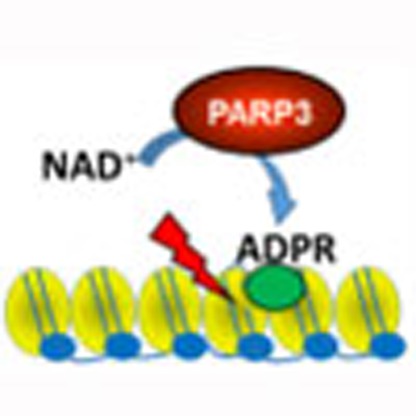

Chromosomal single-strand breaks (SSBs) are the most common DNA lesions arising in cells, arising at a frequency of tens-of-thousands per cell per day^1^,^2^. SSBs can arise directly as a result of oxidative attack of deoxyribose or indirectly as products of topoisomerase activity or DNA base excision repair. The threat posed by unrepaired SSBs is illustrated by the neurological dysfunction observed in individuals in which SSB repair (SSBR) is attenuated^1^,^3^. The detection of at least a subset of SSBs is accelerated by a family of enzymes known as (ADP-ribosyl) transferases (ARTs) that catalyse mono- or poly-ADP-ribosylation; a post-translational modification in which proteins are covalently modified with single or multiple units of ADP-ribose^4^,^5^. Two such enzymes are so far implicated in SSB detection and repair; PARP1 (ADPRT1/ART1), and PARP2 (ADPRT2/ART2). PARP1 is the archetypal SSB sensor and is responsible for most of the cellular poly (ADP-ribosylation) activity following DNA strand breakage^6^,^7^, with PARP2 accounting for 5–15% of this activity and fulfilling a partially overlapping role with PARP1 (refs 8, 9). More recently PARP3 (ADPRT3/ART3) was also implicated in SSB detection by experiments in which nicked oligonucleotide substrates were shown to stimulate PARP3 autoribosylation activity^10^,^11^,^12^,^13^. However, it is not yet known whether PARP3 is involved in sensing SSBs in cells and whether it plays a role in the repair of these lesions. In addition, there is little or no understanding of whether or how chromatin structure affects SSB detection and signalling by ADPRT enzymes. For example, it was established more than 30 years ago that all of the histones in chromatin are ribosylated in permeabilised rat liver nuclei, with histones H1 and H2B the primary targets. However, the identity of the ADPRT enzyme/s responsible for these modifications remains unclear^14^,^15^. Here, we identify the importance, mechanism and role of PARP3 at SSBs and identify for the first time the selective ribosylation of a specific histone at a site-specific DNA break.

## Results

### PARP1 and PARP3 accelerate chromosomal SSBR in DT40 cells

**Figure 1:**
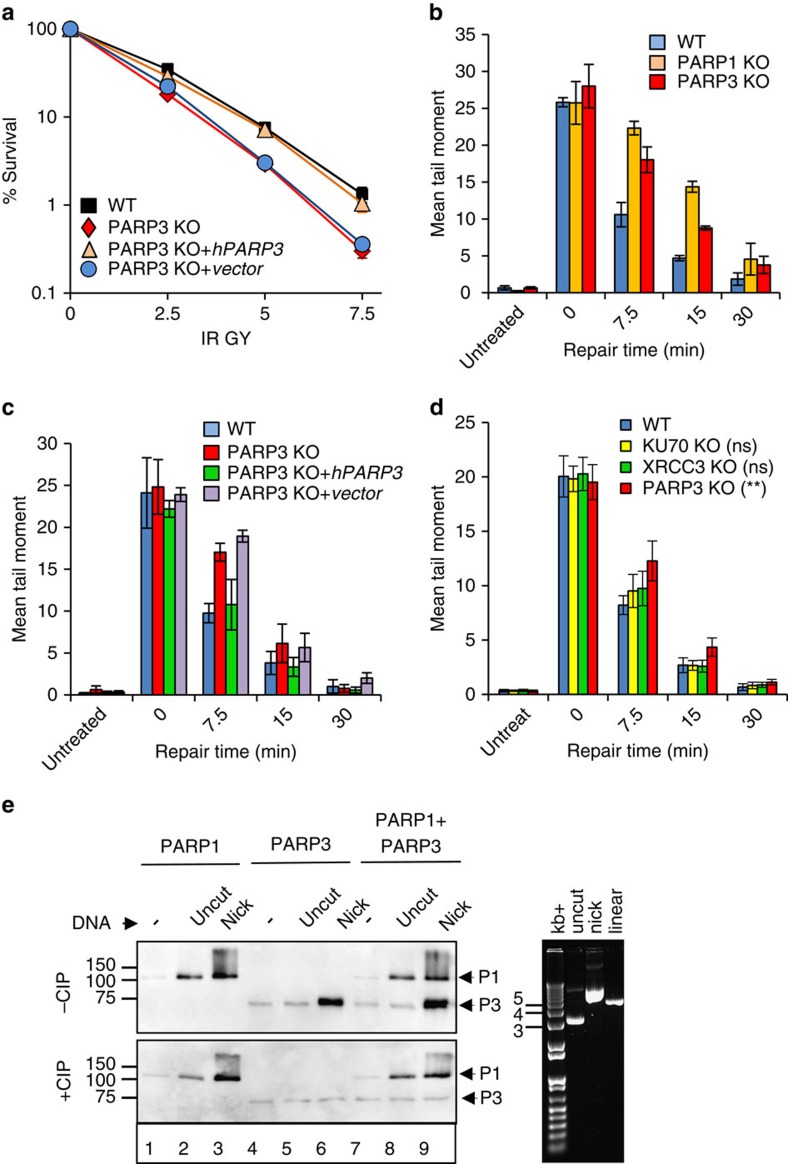
PARP3 promotes chromosomal SSBR and is stimulated by SSBs with canonical termini. (**a**) Wild-type (WT) DT40 cells, *PARP3*^*−/−*^ (PARP3 KO) DT40 cells, and *PARP3*^*−/−*^ DT40 cells stably transfected with either empty vector (vector) or vector encoding human recombinant PARP3 (hPARP3) were treated with the indicated doses of γ-rays and survival calculated in clonogenic assays. Data are the mean (±s.e.m.) of three independent experiments. Where not visible, error bars are smaller than the symbols. (**b**) WT, *PARP1*^*−/−*^, or *PARP3*^*−/−*^ DT40 cells were treated on ice with γ-rays (20 Gy) and incubated for the indicated times to allow repair. DNA strand breaks were quantified (tail moment) by alkaline comet assays. Data are the average tail moment of >50 cells per sample and are the mean of three independent experiments (±s.e.m.). (**c**) WT, *PARP3*^*−/−*^, or derivatives of *PARP3*^*−/−*^ DT40 cells complemented with empty vector or hPARP3 were treated on ice with γ-rays (20 Gy) and incubated for the indicated times to allow repair. DNA strand breaks were quantified as above. (**d**) WT, *KU70*^*−/−*^, or *XRCC3*^*−/−*^ DT40 cells were treated on ice with γ-rays (20 Gy) and incubated for the indicated times to allow repair. DNA strand breaks were quantified as above. ANOVA was employed to compare mutant DT40 for significant differences with WT (**P<0.01. ‘ns'; not significant). Data are the mean (±s.e.m.) of three independent experiments. (**e**) hPARP1 and/or hPARP3 (50 nM) was incubated with 12.5 μM biotin-NAD^+^ and 200 ng uncut or nicked plasmid (nicked with Nt.BsmA1; nick concentration of 32 nM) that was pretreated or not as indicated with CIP to dephosphorylate 5′-termini. Reaction products were separated by SDS–PAGE and blotted with streptavidin-HRP. (right) Aliquots of uncut, nicked, and linear plasmid were analysed by agarose gel electrophoresis and staining with ethidium bromide. ANOVA, analysis of variance; CIP, calf intestinal phosphatise; HRP, horseradish peroxidase

PARP3 is stimulated by DNA double-strand breaks (DSBs) *in vitro* and accelerates the repair of chromosomal DSBs at early times following γ-irradiation by promoting the final step of DNA ligation^10^,^12^. More recently, it was shown that PARP3 is also stimulated by SSBs *in vitro* to a greater extent than DSBs^16^. Since PARP1 is considered the primary SSB sensor protein, we wished to examine whether PARP3 stimulation by SSBs is physiologically relevant by examining the importance of PARP3 for chromosomal SSBR in cells. To do this, we deleted *PARP3* in chicken DT40 cells by targeted gene disruption (Supplementary Fig. 1a). *PARP3*^*−/−*^ DT40 cells were hypersensitive to γ-rays and, similar to *PARP1*^*−/−*^ cells, repaired ionizing radiation-induced DNA-strand breaks more slowly than wild-type cells (Fig. 1a,b). Importantly, both of these phenotypes were corrected by expression of recombinant human PARP3 (Fig. 1a,c). Given that >95% of γ-rays induced DNA breaks are SSBs^17^ these experiments suggest that SSBR is slower in the absence of PARP3. Consistent with this, neither *Ku70*^*−/−*^ or *XRCC3*^*−/−*^ DSB repair-defective DT40 cells^18^,^19^ exhibited significantly slower DNA repair kinetics in alkaline comet assays, suggesting that the level of DSBs induced by γ-irradiation, relative to SSBs, is too low to affect this assay under the conditions employed (Fig. 1d). Collectively, these data suggest that both PARP1 and PARP3 accelerate chromosomal SSBR in avian DT40 cells.

To clarify their respective roles in SSB sensing, we first compared PARP1 and PARP3 for autoribosylation activity in the presence of plasmid substrates containing SSBs. Activation of equimolar amounts of PARP1 and PARP3 by restriction endonuclease-induced nicks was comparable under the conditions employed, suggesting that PARP1 and PARP3 are equally effective sensors of SSBs with canonical 5′-phosphate (5′-P) and 3′-hydroxyl (3′-OH) termini (Fig. 1e; top, lanes 3, 6 and 9). However, consistent with a previous report^16^, PARP3 stimulation was greatly reduced if the DNA nicks harboured 5′-OH termini, whereas PARP1 stimulation was not affected (Fig. 1e; bottom, lanes 3, 6 and 9). Similarly, whereas PARP1 was stimulated by micrococcal nuclease (MNase)-treated plasmid harbouring SSBs and DSBs with non-canonical 3′-P and 5′-OH termini, PARP3 was stimulated only if the 5′- and 3′-termini were restored to canonical 5′-P and 3′-OH moieties (Supplementary Fig. 1b). Collectively, these data suggest that both PARP1 and PARP3 are SSB sensors but that they differ in the types of DNA termini that they can detect.

### Mechanism of DNA break sensing by PARP3

**Figure 2:**
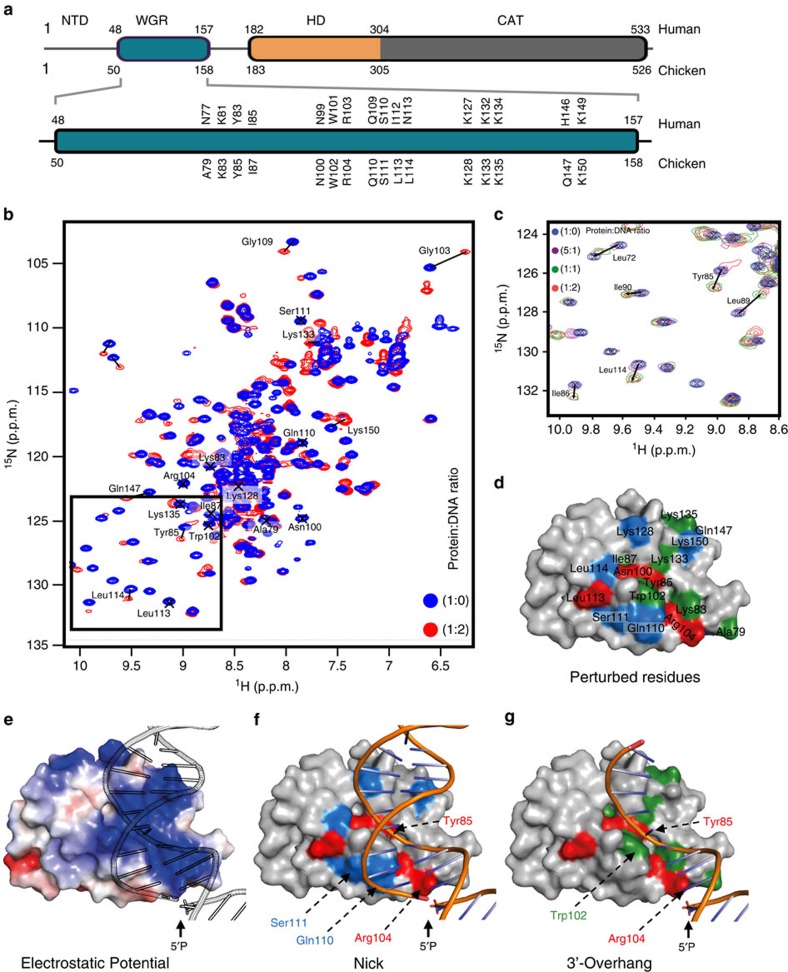
Mechanism of DNA break binding by the cPARP3 WGR domain. (**a**) Schematic representation of PARP3 domains with the human (top) and chicken (bottom) amino acid positions indicated. The WGR domain is shown in expanded format below showing the HSQC perturbed residues of chicken PARP3 (bottom) and the equivalent residues in human PARP3 (top). (**b**) Overlay of ^1^H–^15^N HSQC NMR full spectra for cPARP3^1–169^ in the absence (blue) or presence (red) of oligodeoxyribonucleotide duplex harbouring a 5′-phosphorylated nick (protein:DNA ratios of 1:0 and 1:2, respectively). (**c**) Expanded view of the small boxed region shown in **b** demonstrating the chemical shifts induced in cPARP3^1–169^ by different concentrations of nicked DNA. Protein:DNA ratios were 1:0 (that is, no DNA; blue), 5:1 (magenta), 1:1 (green) and 1:2 (red). (**d**) Map of significant chemical shifts induced in cPARP3^1–169^ by DNA duplex harbouring a 5′-phosphorylated nick (>0.1  p.p.m) or 10-bp 3′-overhang with a recessed 5′-phosphorylated terminus (>0.04), surface modelled using CS-Rosetta^41^. Residues with a significant chemical shift in the presence of either a nick (blue) or 3′-overhang (green) or both (red) are indicated. (**e**) Electrostatic surface of modelled cPARP31–169 with nicked DNA. (**f**) Model of cPARP3^1–169^ with nicked DNA, depicting residues with significant chemical shifts as above. (**g**) Model of cPARP3^1–169^ with nicked DNA lacking the strand located upstream (5′) of the nick (that is, harbouring a DSB with 10-bp 3′-overhang). Residues exhibiting a significant chemical shift are indicated as above.

Human PARP3 (Uniprot accession number Q9Y6F1) consists of three bioinformatically defined regions; a unique N-terminal domain (NTD), a central WGR domain and a C-terminal catalytic domain that includes a helical subdomain (Fig. 2a). To investigate the mechanism of DNA break sensing by PARP3 by nuclear magnetic resonance (NMR), we employed chicken PARP3 (cPARP3; Uniprot accession number, E1BSI0), which is highly similar to hPARP3 in amino acid sequence but is more resistant to proteolysis (Fig. 2a). Two-dimensional (2D) ^1^H–^15^N heteronuclear single quantum coherence (HSQC) NMR spectra of a cPARP3 fragment comprised of the NTD and WGR domain (NTD-WGR: residues 1–169) revealed that while the WGR domain is fully folded, the NTD has poor chemical shift dispersion, suggesting that it is predominantly disordered in solution (Fig. 2b). Nevertheless, ∼160 individual peaks within the NTD-WGR construct were assigned through a combination of standard triple resonance approaches based on HN(CO)CBCA, HNCACB, HNCO and HNCACO experiments. The majority of backbone resonances for the WGR domain were also assigned, allowing us to map in detail its interaction with DNA in detail.

We failed to detect any chemical shift perturbations for residues in the NTD domain in the presence of 19-bp duplex harbouring a 5′-phosphorylated nick, suggesting that this region is not directly involved in DNA binding (Supplementary Fig. 2). Indeed, the majority of residues exhibiting a perturbed chemical shift with nicked DNA mapped to one face of the WGR domain; a conserved and highly basic surface patch that includes the residues Tyr85, Arg104, Lys128 and Lys150 (Fig. 2d–f). Some residues in this patch (for example, Try85 and Arg104) also exhibited chemical shift changes in the presence of a substrate lacking the discontinuous strand upstream of the nick, thus possessing a 10-bp 3′-overhang, suggesting that in the nicked substrate these residues contact the continuous strand of the duplex (Fig. 2d,f,g; red). Interestingly, this 3′-overhang substrate is a DSB of the type preferred by PARP3 and thus most likely relevant to the role of PARP3 in NHEJ^10^,^16^. Indeed, some residues (for example, Trp102) exhibited a shift only with the 3′-overhang DSB substrate (Fig. 2g; green). Conversely, a number of other residues exhibited significant chemical shift changes only with the nicked duplex (for example, Gln110, Ser111, Lys128, Lys150; Fig. 2f, blue) suggesting that these amino acids interact with DNA duplex on the 3′ side of the nick and are important specifically for SSB sensing. Together these data allowed the construction of docked structural models for the WGR domain of cPARP3 bound to a DNA break in which Tyr85 is located in a valley that contacts continuous DNA backbone and Arg104 is positioned close to the 5′-phosphorylated terminus, directly implicating this residue in ‘sensing' the 5′-phosphate. We did not detect any significant perturbations in the cPARP3 WGR domain if the DNA substrates lacked a 5′-phosphate.

**Figure 3:**
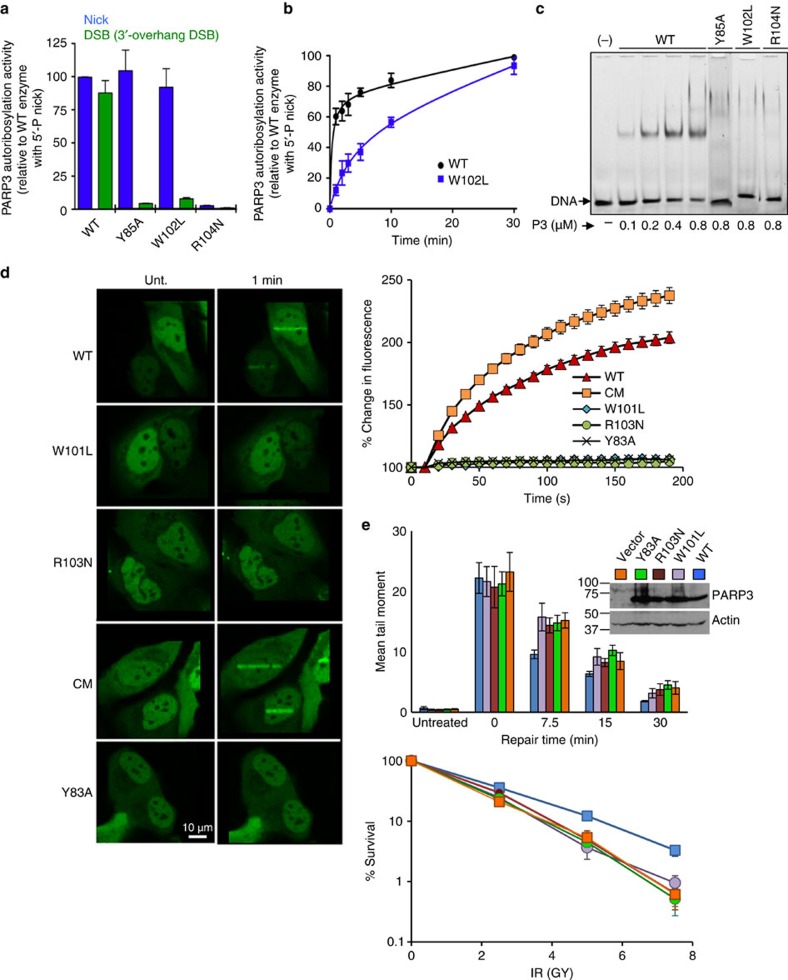
The PARP3 DNA-binding interface is required for PARP3 stimulation and accumulation at chromosome DNA damage. (**a**) Wild-type or the indicated mutant full-length cPARP3 (300 nM) was incubated for 20 min at room temp with biotin-NAD^+^ (12.5 μM) and 200 nM of oligonucleotide duplex harbouring either a 5′-phosphorylated nick or 5′-phosphorylated DSB with 3′-overhang. Reaction products were separated by SDS–PAGE, blotted, and detected with streptavidin-HRP. Autoribosylated cPARP3 was quantified and plotted relative to that generated in reactions containing nicked duplex and wild type cPARP3. Data are the mean (±s.e.m.) from three independent experiments. (**b**) Time-course of wild-type or mutant cPARP3 incubated from 0 to 30 min in the same conditions as above. (**c**) Recombinant wild-type or mutant cPARP3 (0–0.8 μM) was incubated with a 3′-fluorescein isothiocyanate (FITC)-labeled oligonucleotide duplex harbouring a 5′-phosphorylated nick (100 nM), and protein-DNA complexes detected by EMSA. (**d**) Recruitment of wild-type and mutant human PARP3-GFP to sites of UVA-laser DNA damage in human U2-OS cells. (left) Representative images of WT and mutant PARP3-GFP before treatment (Unt) and 1 min after laser damage. (top right) Quantification of GFP accumulation at sites of laser damage (% increase over initial level). Data are the mean (±s.e.m.) of 25 or more cells per sample. The hPARP3 WGR mutations were Y83A, W101L and R103N and H384A/E514A in the catalytic domain (denoted ‘CM'). (**e**, top) *PARP3*^*−/−*^ DT40 cells stably transfected with either empty vector (vector) or vector encoding wild-type hPARP3 (WT) or the mutant derivatives Y83A, W101L and R103N were treated on ice with γ-rays (20 Gy) and incubated for the indicated times to allow repair. DNA strand breaks were quantified (tail moment) by alkaline comet assays. The inset is a western blot showing the expression level of wild type and mutant hPARP3 in *PARP3*^*−/−*^ DT40 cells. (bottom) The above DT40 cell lines were treated with the indicated doses of γ-rays and survival quantified in clonogenic assays. Data are the mean (±s.e.m.) of three independent experiments. Where not visible, error bars are smaller than the symbols. HRP, horseradish peroxidase.

To test aspects of this model we mutated residues predicted to be important for WGR function in chicken and human PARP3 and, after confirming they did not reduce protein stability (Supplementary Fig. 3), examined their impact on PARP3 function. Consistent with its putative role in 5′-phosphate ‘sensing', mutation of Arg104 (R104N) greatly reduced or ablated stimulation of chicken PARP3 (cPARP3) both by nicked duplex and by the 3′-overhang DSB substrate (Fig. 3a). In contrast, although mutation of Trp102 (W102L) prevented cPARP3 stimulation by the 3′-overhang DSB substrate it only slowed cPARP3 activity on the nicked duplex (Fig. 3a,b). This suggests that the DNA contact made by W102 promotes but is not essential for cPARP3 activity on nicked DNA because of compensatory contacts with duplex DNA on the 3′ side of the nick. Tyr85 (Y85A) similarly prevented cPARP3 stimulation by the 3′-overhang substrate and had little impact on the nicked substrate, suggesting that this residue is also more important for the 3′-overhang DSB substrate (Fig. 3a). In contrast, however, all three of the WGR mutations prevented both detectable binding of cPARP3 to nicked DNA in electrophoretic mobility shift assays (EMSAs) and the accumulation of human PARP3 (hPARP3) at sites of ultraviolet A (UVA)-laser damage in cells. This suggests that some WGR mutations allow sufficient interaction with nicked DNA duplex to slowly stimulate PARP3 activity but insufficient interaction for stable DNA binding (Fig. 3c,d). Consistent with this, all three of the WGR mutations greatly reduced or ablated the ability of hPARP3 to correct the defects in DNA-strand break repair and cell survival in *PARP3*^*−/−*^ DT40 cells, following γ-irradiation (Fig. 3e).

### PARP3 preferentially monoribosylates H2B in nicked chromatin

**Figure 4:**
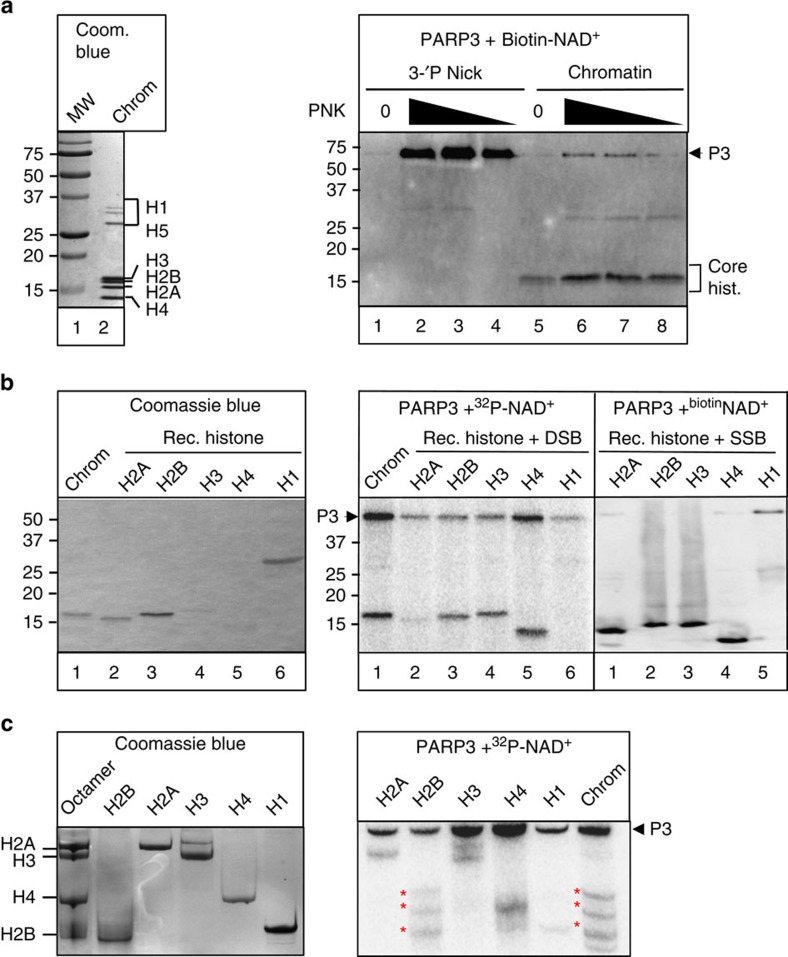
PARP3 monoribosylates H2B in damaged chromatin. (**a**, left) 10μg of the chicken chromatin employed in these experiments was fractionated by SDS–PAGE and stained with Coomassie blue. (right) One microgram of soluble MNase-treated chicken chromatin or 50-mer oligonucleotide duplex (200 nM) harbouring a nick with 3′-P/5′-OH termini was mock-treated (0) or treated with 1, 0.5 or 0.25 U T4 PNK to restore 3′-OH/5′-P termini. These DNA substrates were then incubated with 100 nM hPARP3 and 12.5 μM biotin-NAD^+^ for 30 min and biotinylated products separated by 15% SDS–PAGE and detected with streptavidin-HRP. (**b**) 1 μg chicken chromatin or the indicated recombinant histone was incubated with 100 nM hPARP3 in the presence of 300 nM ^32^P-NAD^+^ or 12.5 μM biotin-NAD and oligonucleotide harbouring either a DSB (middle) or SSB (right) and the reaction products fractionated by 15% SDS–PAGE and detected by autoradiography or streptavidin-HRP. (left) An aliquot of the chicken chromatin and recombinant histones was fractionated by SDS–PAGE and stained with Coomassie blue. (**c**, left) Aliquots of recombinant histone standards were fractionated separately or together as an octamer on triton-acid urea gels and analysed by staining with Coomassie blue. (right) The products of the PARP3 ribosylation reactions conducted in **b** were fractionated on triton-acid urea gels and analysed by autoradiography. HRP, horseradish peroxidase.

Given the ability of PARP3 to bind damaged chromatin we examined whether chromatin is a target of PARP3 activity. Indeed, whereas PARP3 efficiently autoribosylated itself in the presence of nicked oligonucleotide DNA duplex it preferentially transribosylated one or more core histones in the presence of micrococcal nuclease (MNase)-treated chicken chromatin (Fig. 4a). As expected this stimulation was largely dependent on pre-treatment of the chromatin with T4 PNK, confirming that the PARP3 preference for canonical termini observed in naked DNA was retained in chromatin.

To identify the histone/s ribosylated by PARP3, we compared the ribosylation product in chicken chromatin with that of individual recombinant histones. In contrast to chicken chromatin all four recombinant core histones were ribosylated by PARP3 in the presence of DSBs or DNA nicks, suggesting that chromatin structure confers substrate specificity on PARP3 thereby targeting the enzyme to a single-histone subunit (Fig. 4b, right). A comparison of the electrophoretic mobility of the ribosylated histone in chicken chromatin with that of recombinant histones suggested that the former was either H2B or H3 (Fig. 4b, middle and right). Indeed, subsequent fractionation of the ribosylated chromatin proteins on triton-acid urea gels revealed a banding pattern that closely resembled the banding pattern of ribosylated H2B (Fig. 4c, red asterisks). It is currently unclear what the different ribosylated isoforms of H2B represent but these data clearly implicate H2B as the target of PARP3 ribosylation at DNA breaks. In contrast, PARP1 preferentially transribosylated the linker histones H1 and H5 in chicken chromatin, suggesting that these two SSB sensor proteins differ in their chromatin protein targets (Supplementary Fig. 4a). Importantly, the ADP-ribose moiety on H2B was sensitive both to hydroxylamine and MacroD1 (refs 20, 21) but was insensitive to PARG, collectively suggesting that this modification was mono (ADP-ribose) and was located on an acidic amino acid (Supplementary Fig. 4b,c). This contrasted with PARP1, which as expected generated poly (ADP-ribose) products that were sensitive to PARG but not MacroD1 (Supplementary Fig. 4d).

### PARP3 binds mononucleosomes and ribosylates H2B^E2^

**Figure 5:**
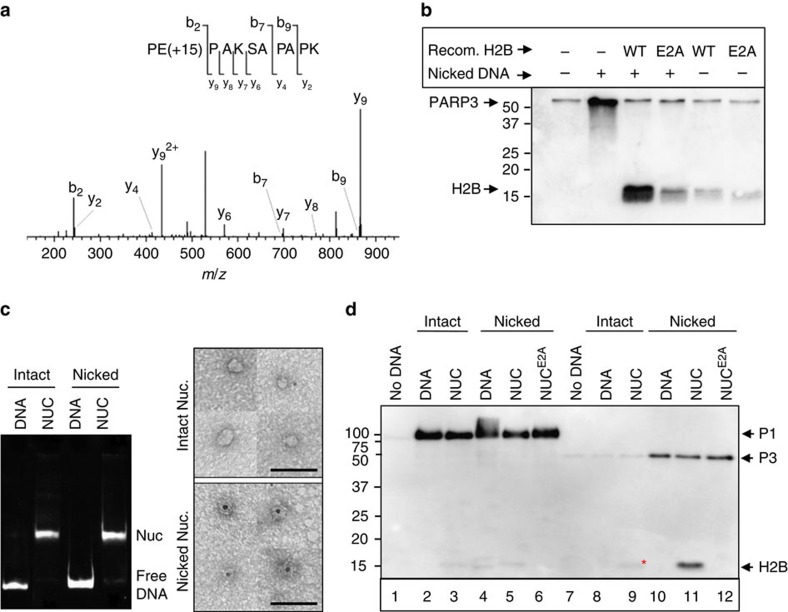
PARP3 binds nicked nucleosomes and ribosylates H2B^E2^. (**a**) ADP-ribosylation of E2 in recombinant H2B by hPARP3. MS/MS fragmentation profile of doubly charged PE(+15)PAKSAPAPK (theoretical: 554.3115 *m/z*; observed: 554.3113 *m/z*; 0.4 p.p.m. mass error), indicating the position of the NH (+15.0109 Da) moiety on glutamate resulting from hydroxylamine derivatization of ADP-ribose. Preferred fragmentation N-terminal to prolines is observed, as expected^43^. (**b**) Mutation of E2 reduces ribosylation of H2B by hPARP3. The products of ribosylation reactions containing 200 nM hPARP3, 12.5 μM biotin-NAD^+^, 100 nM nicked oligonucleotide duplex (50 bp), and 5 μM of wild-type or mutant recombinant H2B were separated on 15% SDS–PAGE gels and detected by autoradiaography. (**c**) PARP3 binds to nicked mononuclesomes. (left) Reconstituted mononucleosomes were assembled on intact or nicked DNA (Widom positioning sequence 601.2) and nucleosome quality assessed by native gel electrophoresis. (right) representative images of negative stained intact (top) or nicked (bottom) nucleosomes incubated with His-tagged cPARP3, and with the His-tagged protein detected by nanogold Ni-NTA. Scale bars, 50nm. (**d**) PARP3 ribosylates H2B^E2^ in reconstituted nicked mononucleosomes. Hundred nanomolar of intact or nicked 601.2 DNA, present either as naked duplex or within reconstituted nucleosomes containing wild-type or mutant H2B, was incubated with 100 nM hPARP1 (lanes 1–6) or hPARP3 (lanes 7–12) and either 12.5 μM biotin-NAD^+^ (hPARP3) or 1.5 μM biotin-NAD^+^ (hPARP1, to encourage shorter chain modifications). MS/MS, tandem mass spectrometry.

To identify the ribosylation site on H2B the recombinant histone was incubated with PARP3 and the modified tryptic peptides enriched by boronate affinity chromatography. The affinity-enriched peptides were then derivatized with hydroxylamine to convert ribosylated glutamate/aspartate side chains to hydroxamic acid. Mass spectrometric analysis detected the expected +15.0109 Da shift for the hydroxamic acid derivative on two peptides; PEPAK and PEPAKSAPAPK (Fig. 5a), unequivocally identifying E2 as a modified residue. This did not reflect nonspecific derivatization of E2 because the modified peptide was highly enriched by boronate affinity chromatography and was undetectable if H2B was not pretreated with PARP3 (Supplementary Fig. 5a). This was in contrast to E105 (LLLPGELAK), which was not enriched by boronate chromatography and was derivatized independently of pretreatment with PARP3 (Supplementary Fig. 5b). More importantly, ribosylation of H2B by PARP3 was greatly reduced if we employed recombinant H2B in which E2 was mutated to alanine (H2B^E2A^), confirming this residue as the primary site of ribosylation by PARP3 (Fig. 5b).

Next, we packaged intact DNA or DNA harbouring a site-specific nick in recombinant mononucleosomes; the physiologically relevant structures in chromatin in which SSBs occur (Fig. 5c, left). PARP3 bound nicked nucleosomes with much greater affinity than intact nucleosomes as measured by indirect immunogold labelling of PARP3 and electron microscopy with a gold particle co-locating with 30 and 85% of intact and nicked nucleosomes, respectively (Fig. 5c, right). That we observed only single gold particles on nicked nucleosomes strongly suggests that PARP3 bound as a monomer. To our knowledge, this is the first demonstration of nucleosome binding by a PARP enzyme in DNA strand break-specific manner. Moreover, in agreement with our experiments using bulk chicken chromatin, PARP3 preferentially ribosylated a single-core histone in the nicked nucleosomes (Fig. 5d, ‘lane 11'). This event was greatly reduced if we employed either intact nucleosomes lacking the site-specific nick (Fig. 5d, ‘lane 9') or nicked nucleosomes containing mutant H2B^E2A^ (Fig. 5d, ‘lane 12'), confirming both that PARP3 preferentially ribosylates H2B^E2^ in mononucleosomes and that it does so in response to the site-specific SSB. We noted that a small amount of ribosylated H2B was also present in intact nucleosomes (Fig. 5d, lane 9, asterisk), presumably triggered by weak stimulation of PARP3 by the DSB ends of the nucleosome DNA. In contrast, PARP1 ribosylated mononucleosomes only very weakly even in the presence of the site-specific SSB, confirming the specificity of the ADP-ribosylation reaction by PARP3 (Fig. 5d, ‘lanes 2–6').

## Discussion

Protein ADP-ribosylation is a post-translational modification that regulates various biochemical processes and is catalysed by a superfamily of proteins known as (ADP-ribosyl) transferases (ADPRTs)^5^. To date, three ADPRTs are implicated in DNA damage signalling; PARP1, PARP2 and PARP3 (ref. 22). These enzymes detect DNA strand breaks by binding these lesions and becoming catalytically activated or stimulated^23^ and thereby ribosylating themselves (autoribosylation) and/or other proteins (trans-ribosylation) including histones^14^,^24^,^25^,^26^. However, the specificity of these modifications for a particular type of lesion and/or a specific ADPRT is unclear. Here, we have identified H2B^E2^ as a specific target of PARP3 both in purified chicken chromatin and in reconstituted recombinant nucleosomes in response to SSBs. The targeting of H2B in both synthetic nucleosomes and chicken chromatin suggests that this ribosylation event occurs independently of the sequence context or position of the nick. Indeed, we also observed H2B ribosylation if we assembled nucleosomes on a 246-bp mouse rDNA sequence, in which the nick was located at a different position (Supplementary Fig. 6). It remains possible however that the efficiency of the ADP-ribosylation reaction is affected by the position of the nick.

While DNA nicks stimulate PARP3 to a greater extent than DSBs, we noted that H2B ribosylation by PARP3 also occurred at DSBs. This was suggested by the ribosylation of recombinant H2B by PARP3 in the presence of non-nicked oligonucleotide duplexes (see Fig. 4b) and by the small amount of H2B ribosylation observed in reactions containing intact (that is, non-nicked) nucleosomes, triggered most likely by the ends of the nucleosome DNA sequence (see Fig. 5d). That H2B was ribosylated to a greater extent by SSBs than DSBs is consistent with the relative activity of PARP3 on these two types of DNA break. The NMR experiments conducted here provide a model for binding of PARP3 both to a DNA nick and to a 3′-overhang; a type of DSB favoured by this enzyme^10^. Mutation of key residues predicted by NMR to be important for binding these DNA breaks reduced PARP3 activity *in vitro*, to a greater or lesser extent, and disrupted stable binding to DNA in EMSAs. In contrast to these data, Langelier *et al.* did not detect an impact of the WGR residue W101 on DNA binding by human PARP3, yet detected more of an impact than us on activity (Fig. 3b and ref. 16). We believe these discrepancies are due to the different techniques and ionic conditions employed in the two studies. For example, the fluorescence polarization assays employed by Langelier *et al.* are less sensitive to defects in stable binding than are the EMSAs we employed, but the lower ionic concentrations we employed allow more activity. Finally, that the structural models predicted here are physiologically relevant was suggested by our observation that human PARP3 harbouring the WGR mutations was unable to accumulate at sites of UVA-laser damage or to accelerate DNA strand break repair following ionizing radiation.

H2B^E2^ was identified more than 30 years ago as monoribosylated in rat liver nuclei *in vitro*, with ∼15% of total H2B ribosylated when such nuclei are incubated with [^14^C]NAD^+^ (ref. 14). However, the identity of the ADPRT responsible for this modification has remained elusive, as has the molecular trigger for this ADP-ribosylation event. Our data provide compelling evidence that PARP3 is a major source of this modification, particularly in response to SSBs, though whether PARP3 is the only source of this modification remains to be determined. Nevertheless, to our knowledge these data are the first report of a site-specific chromatin modification by a PARP enzyme in response to a specific DNA lesion.

The archetypal SSB sensor protein is PARP1 (refs 4, 27). However, both PARP1 and PARP3 accelerated the repair of γ-ray induced SSBs in chicken DT40 cells in our experiments. It will be of interest to determine if this is also true in human cells, which unlike DT40 cells additionally possess a PARP2 enzyme that might functionally overlap with PARP3. Consistent with this possibility, similar to PARP3, PARP2 is sensitive to the chemistry of DNA termini^16^ and in our experiments overexpression of PARP2 partially corrected the reduced DNA strand break repair rate and resistance to γ-rays observed in *PARP3*^*−/−*^ DT40 cells (Supplementary Fig. 7). PARP1 and PARP3 were both activated if incubated together and did not inhibit each other even in the presence of sub-stoichiometric amounts of nicked DNA raising the possibility that both enzymes are activated by the same DNA breaks. However, since PARP1 and PARP3 exhibit different DNA termini specificities they most likely also fulfil spatially or temporally distinct roles. For example, perhaps PARP1 and PARP3 sense SSBs sequentially during SSBR, detecting the breaks before and after canonical 3′-hydroxyl and 5′-phosphate have been restored. Alternatively, perhaps PARP1 and PARP3 are components of different SSBR processes, detecting SSBs in different subcellular contexts.

In addition to being stimulated by different types of SSB, PARP1 and PARP3 prefer different chromatin targets. PARP1 primarily ribosylates itself and histone H1 (refs 28, 29), whereas PARP3 preferentially modifies itself and histone H2B^E2^. The structure of the modifications is also distinct with PARP1 synthesizing primarily poly (ADP-ribose) and PARP3 primarily modifying proteins with mono (ADP-ribose). The role of this modification is unknown but it is unlikely that mono (ADP-ribose) significantly affects the structure of nucleosomes directly. Indeed, ribosylated H2B co-fractionated with the mononucleosomes during gel filtration suggesting that the ribosylated nucleosomes were intact (Supplementary Fig. 8). It will be of interest to determine whether PARP3 ribosylates only the nucleosome in which the DNA break is located or whether it can function in *trans*, thereby ribosylating distal nucleosomes. In our experiments ribosylation of H2B was inefficient in intact nucleosome that were incubated with stoichiometric amounts of naked nicked DNA, suggesting that ADP-ribosylation occurred in *cis* under these experimental conditions, thereby marking the site of the break (Supplementary Fig. 9). However, whether ribosylation can also occur in *trans* if undamaged nucleosomes are present in close proximity or at high concentration remains to be determined. Nevertheless, irrespective of whether H2B ribosylation occurs in *cis* and/or in *trans*, it will now be of interest to identify the protein/s that detect mono ADP-ribosylated histone H2B and translate this modification into a biological function.

In summary, we show here that PARP3 is stimulated by SSBs *in vitro* and is required for normal rates of chromosomal SSBR in chicken DT40 cells. PARP3 preferentially binds to and is activated by nicked mononucleosomes, which target PARP3 trans-ribosylation activity to a single-histone substrate. Although nicks in naked DNA stimulate PARP3 autoribosylation, nicks in mononucleosomes promote preferential trans-ribosylation of histone H2B^Glu2^. These data identify PARP3 as a molecular sensor of nicked nucleosomes and demonstrate, for the first time, the site-specific ribosylation of chromatin at a defined DNA single-strand break.

## Methods

### DNA substrates

pEGFP (500 ng) was digested or not with Nt.BsmA1 (20 U) and then mock-treated or treated with 10 U CIP (New England Biolabs). Following purification (QIAquick spin column; Qiagen), the DNA was incubated with different concentrations of micrococcal nuclease (MNase)(Worthington) in 50 mM Tris-HCl 7.5, 5 mM CaCl_2_ and 0.1 mg ml^−1^ bovine serum albumin for 30 min at room temperature. Reactions were stopped with EDTA (50 mM final) and the DNA purified as above. DNA from the MNase concentration that produced the greatest SSB/DSB ratio (0.015 U) was mock-treated or treated with T4 PNK in the presence of 2 mM ATP and 10U T4 PNK enzyme (wild-type or 3′-phosphatase dead; New England Biolabs). The synthetic oligonucleotide sequences (MWG or Eurogentec) employed to generate duplex substrates are listed in Supplementary Table 1.

### Recombinant proteins

Untagged full-length human PARP1 (hPARP1) and N-terminal His-tagged PARP3 (hPARP3) were expressed using baculovirus in Sf9 cells and purified by 3-aminobenzamide and Ni-agarose affinity resins, respectively^10^,^30^. Both proteins were further purified by gel filtration in 20 mM Tris-HCl, 0.3 M NaCl, 5% (v/v) glycerol, 1 mM DTT, and frozen in aliquots at −80 °C. PARG protein was obtained from AMS Biotech (Abingdon, UK). A synthetic codon-optimized gene for expression in *Escherichia coli,* encoding full-length *Gallus gallus* PARP3 (chicken PARP3; cPARP3) was purchased from GenScript (Piscataway, NJ, USA). The cPARP3 open reading frame (ORF) was amplified by PCR using the primers cPARP3-pNIC28-Fw and cPARP3-pNIC28-Rv (for full-length cPARP3) or cPARP3-pNIC28-Fw and cPARP3-pNIC28-169Rv (for cPARP3^1–169^) and inserted into the vector pNIC28-Bsa4 by ligation-independent cloning^31^ (Supplementary Table 1). Site-specific mutations were introduced using a Quickchange site-directed mutagenesis kit (Stratagene) using the oligonucleotides indicated in Supplementary Table 1. cPARP3 proteins were expressed in the *E. coli* strain Rosetta2(DE3)pLysS (Merck Chemicals, Nottingham, UK) in Turbo-broth (Molecular Dimensions, Newmarket, UK). Protein expression was induced by the addition of 0.3 mM IPTG at a culture OD_600_ of 2 and the growth temperature reduced from 37 °C to 18 °C for induction overnight (22–24 h). For NMR experiments, the protein was expressed in 3 l filter-sterilized Overnight Express Autoinduction NMR Media (Merck-Millipore, Billerica, MA, USA) containing 50 mM [^15^N] NH_4_Cl and 0.5% (w/v) [^13^C_3_] glycerol (CortecNet, Voisins-le-Bretonneux, France) at a temperature of 25 °C for 24 h. The resulting cell pellets were resuspended in 50 ml Buffer A (50 mM HEPES-HCl pH 7.5, 0.5 M NaCl, 0.5 mM TCEP) supplemented with protease inhibitors (Roche, Burgess Hill, UK), lysed by sonication, and the clarified supernatant applied to a 5 ml Talon (TaKaRa Bio, Saint-Germain-en-Laye, France) affinity column by gravity flow in Buffer A. After successive washes in Buffer A (20 CV), bound protein was eluted in Buffer B (50 mM HEPES-HCl pH 7.4, 0.5 M NaCl, 0.5 mM TCEP, 0.3 M imidazole) and eluted cPARP3 concentrated using Vivaspin 20 (30,000 MWCO) centrifugal concentrators (Sartorius Stedim Biotech, Goettingen, Germany). cPARP3 was diluted fivefold in Buffer C (50 mM HEPES-HCl pH 7.5, 150 mM NaCl, 0.5 mM TCEP) and further purified on a 5 ml HiTrap Heparin HP column (GE Healthcare, Little Chalfont, UK) using a linear salt gradient. Fractions containing cPARP3 were pooled, concentrated, incubated (unless otherwise indicated) with TEV protease to remove the His-tag, and finally applied to a HiLoad Superdex 200 size-exclusion column (GE Healthcare) with a 1 ml HisTrap HP column connected in-line in Buffer D (20 mM HEPES-HCl pH 7.5, 250 mM NaCl, 0.5 mM TCEP). Fractions containing purified cPARP3 were again pooled, concentrated to 20 mg ml^−1^, and either stored at 4 °C for immediate use or flash-frozen in liquid nitrogen and stored at −80 °C until required. The human histone H1.2 ORF was subcloned from IMAGE clone 3608862 into the *Nco*I and *Xho*I sites of pET16b and expressed in BL21(DE3) cells. Inclusion bodies were solubilised in 6 M guanidine HCl, 25 mM Tris-HCl pH 8.0, 1 mM DTT for 1h at room temperature and then refolded by dialysis in 10 mM Tris-HCl pH 7.5, 2 M NaCl, 1 mM EDTA, 1 mM DTT, and purified by gel filtration in the above refolding buffer. IMAGE clone 3349763, containing the MacroD1 cDNA was obtained from Source Bioscience (Nottingham, UK), and the ORF was subcloned into the *Nde*I and *Xho*I sites of pET16b. Protein was expressed in BL21 (DE3) overnight at 16 °C in the presence of 1 mM IPTG, and soluble protein was purified by Ni-NTA affinity chromatography and gel filtration (Superdex 200) in 20 mM Tris-HCl pH 7.5, 0.3 M NaCl, 5% glycerol, 1 mM DTT. *Xenopus laevis* H2A, H3 and H4 histones were expressed and purified from *E. coli* as described^32^. Full length H2B was inserted into pET3a using the primers H2BF, and H2BR (Supplementary Table 1). Alternatively, human H2B expressed from pET28a-hH2B.1 (a gift from Joe Landry; Addgene plasmid #42630) was employed (Fig. 5b,e and Supplementary Fig. 9) and the E2A mutant generated by site-directed mutagenesis using pET28a-H2B-E2AF and pET28a-H2B-E2AR (Supplementary Table 1).

### Preparation of soluble chicken chromatin

Chicken blood was collected at slaughter into an equal volume of PBS, 10 mM sodium butyrate, 5 mM EDTA, 0.1 mM phenylmethylsulphonyl fluoride (PMSF) and 0.1 mM benzamidine, and filtered through six layers of cotton gauze. Erythrocytes were pelleted (3,000*g* for 6 min) and the buffy coat removed by pipetting. Erythrocytes were washed by resuspension in collection buffer minus EDTA, re-pelleted as above, and stored at −80 °C until required. On thawing, one volume of cell lysis buffer (CLB; 80 mM NaCl, 10 mM Tris-HCl pH7.4, 6 mM MgCl_2_, 0.1% Triton X-100, 0.1 mM PMSF and 0.1 mM benzamidine) was added and the suspension added to 2 l CLB buffer at 4 °C and stirred for 20 min. Nuclei were pelleted by centrifugation 3,800*g* for 15 min in a swing-out rotor (no brake). Pellets were washed again in NWB, centrifuged as above, and resuspended in 35 ml NWB in a 50 ml Falcon tube. The suspension was underlayed with 15 ml 30% (w/v) sucrose containing 1 × NWB and immediately centrifuged (2,400*g* for 10 min at 4 °C). The final nuclear pellet was resuspended in a minimum volume of NWB, mixed with an equal volume of 80% glycerol, 1 × NWB, frozen and stored at −80 °C. Nuclear pellets (∼40 mg DNA) were mixed with five volumes of digest buffer (DB; 10 mM Tris-HCl pH7.4, 150 mM NaCl, 0.25 M sucrose, 0.3 mM CaCl_2_, 0.1 mM PMSF and 0.1 mM benzamidine) and pelleted by centrifugation (2,000*g* for 7 min at 4 °C). The nuclear pellets were resuspended in 15 ml DB, re-pelleted as above, and finally resuspended in 6 ml DB to give a final DNA concentration of 6 mg ml^−1^. 400 U ml^−1^ micrococcal nuclease (Worthington) was added to pre-warmed nuclei for 20 s at 37 °C and the reaction terminated by addition of EDTA to a final concentration of 10 mM and cooled on ice. An equal volume of lysis buffer (LB; 10 mM Tris-HCl pH7.4, 150 mM NaCl, 0.25 M sucrose, 2 mM EDTA, 0.1 mM PMSF and 0.1 mM benzamidine) was added, and the supernatant (S1) containing the solubilized chromatin clarified by centrifugation (2,000*g* for 7 min). The pellet was resuspended in a further 5 ml LB and centrifuged as above to recover S2. This process was repeated a further four times to recover supernatants S3 to S6. The supernatants were then pooled and clarified a final time by centrifugation (25,000*g* for 20 min), and the chromatin fragments separated by centrifugation on 30 ml sucrose gradients in 20 mM Tris-HCl pH7.4, 50 mM NaCl, 0.1 mM EDTA, 0.1 mM PMSF, 0.1 mM benzamidine, and prepared using an exponential gradient maker, in which the mixing chamber (25 ml) contained 5% w/w sucrose and the reservoir contained 40% sucrose. 3 ml of solubilized chromatin was layered onto each gradient and fractionated by centrifugation at 141,000*g* for 2 h at 4 °C in a Beckman SW28 rotor. Gradients were fractionated by upward displacement using FC-43 (3 M) in an Isco gradient fractionator collecting 1.4 ml fractions. DNA size distributions in the chromatin fractions were assessed by agarose gel electrophoresis and histone integrity by SDS–polyacrylamide gel electrophoresis (SDS–PAGE). Chromatin fragments were stored at −80 °C in sucrose gradient buffer.

### Nucleosome reconstitution

Following removal of DNA using hydroxyapatite, histone octamers were assembled using equimolar amounts of recombinant histones (denatured in 6 M Guanidine HCl, 25 mM Tris-HCl pH 8) by overnight dialysis in 10 mM Tris-HCl, 2 M NaCl, 1 mM EDTA, 1 mM DTT and purified on gel filtration using the same buffer. The 601.2 nucleosome positioning element^33^,^34^ was prepared from 0.1 mg of pUC19-16x601.2 (023) (ref. 35) containing 16 copies of 601.2, by overnight digestion with *Eco*RI and *Eco*RV (NEB). The gel-purified fragment (181 bp, plus a 4 bp 5′-overhang) was dephosphorylated with CIP and, where appropriate, incubated with the nicking enzyme Nt.BsmAI to introduce a single nick at nucleotide 45. Small-scale nucleosome assembly was performed by mixing the DNA and histone octamer (1:1 molar ratio) and reducing the concentration gradually by dialysis from 2 M NaCl to 0.2 M NaCl (ref. 32). The quality of the nucleosome preparation (final conc, 100–300 nM) was assessed by native gel electrophoresis (4.5% 37.5:1 acrylamide: bisacrylamide in 0.4X Tris Boronate EDTA buffer) and staining with ethidium bromide. Where indicated, we also assembled mononucleosomes on a 246-bp mouse 45 S rDNA-positioning element, (Supplementary Fig. 6), prepared by PCR and gel purified^36^.

### PARP assays

PARP autoribosylation assays were performed as described in the figure legends with the indicated PARP1/PARP3 and DNA substrates in the presence of 12.5 μM biotinylated NAD^+^ (6-Biotin-17-NAD; AMS biotech) and in 50 mM Tris-HCl pH 7.5, 50 mM NaCl, 0.5% glycerol, 0.1 mM DTT. PARP assays containing chromatin (0.1 mg ml^−1^) or recombinant histones (0.1 mg ml^−1^) were performed in chromatin storage buffer (20 mM Tris-HCl pH7.4, 50 mM NaCl, 0.1 mM EDTA, 20% sucrose) to maintain chromatin structure. Assays containing reconstituted nucleosomes were performed in 20 mM Tris-HCl pH 7.5 and 0.2 M NaCl. Reactions were stopped with SDS-loading buffer and reaction products fractionated on either 10% or 15% (for analysis of histones) Tris-glycine SDS–PAGE gels or, in the case of reactions conducted with cPARP3, on NuPAGE Novex 4–12% Bis-Tris SDS–PAGE Gels (Invitrogen, Waltham, Massachusetts, USA). Where indicated, Triton-Acid Urea gels were also employed^37^. Note that in our experiments, H4 migrated more slowly than H2B, most likely in part because of the higher triton concentrations employed^38^. For quantification of PARP activity fractionated proteins were transferred to nitrocellulose filters that were blocked in 2% bovine serum albumin/Tris Buffered Saline with Tween 20 (TBST) and probed with streptavidin-HRP (Pierce; 1:30,000 dilution) in blocking solution. Gel bands were quantified using LAS4000 and ImageQuantTL software (GE Healthcare). Where indicated, 300 nM ^32^P-NAD (Perkin Elmer) was employed instead of biotinylated NAD^+^ and SDS–PAGE gels stained with Coomassie Blue before drying and analysis on a phosphorimager.

### Boronate affinity chromatography and mass spectrometry

Products of ADP-ribosylation reactions containing 10 μg of protein were purified using standard TCA precipitation and resuspended in PBA buffer (100 mM HEPES pH 8.5, 150 mM NaCl, 2 mM MgCl_2_). If required, cysteines were reduced in 10 mM dithiotreitol for 30 min at room temperature and alkylated with 10 mM iodoacetamide for 30 min at room temperature in the dark. Proteins were digested with 100 ng trypsin (Promega) overnight at 37 °C and peptides bound to m-aminophenylboronic acid agarose beads (Sigma) for 1 h at 4 °C. The beads were washed extensively with PBA buffer and the ADP-ribosylated peptides eluted with 1 M hydroxylamine (Sigma) pH 7.0 overnight at room temperature. Eluates were purified using C_18_ ZipTips (Millipore) according to manufacturer instructions and analysed by nano-LC-MS (ThermoFisher U3000 nanoLC and Orbitrap XL mass spectrometer)^39^. The raw mass spectrometry and tandem mass spectra were converted to.mgf format using Compass^39^ and searched against the SwissProt database using Mascot (Matrix Science). Search parameters employed a precursor tolerance of 7 p.p.m. and a fragment ion tolerance of 0.8 Da. Quantification of precursor ions employed Skyline^40^. ADP-ribosylation sites were identified based on a characteristic +15.0109 Da shift on glutamate and aspartate residues^41^.

### NMR resonance assignment

NMR spectra were recorded at 303 K on Bruker DRX600 and DRX800 spectrometers equipped with cryo-probes. cPARP3^1–169^ was dissolved in 300 μl NMR buffer containing 20 mM Tris-HCl, pH 7.0, 125 mM NaCl, 1 mM TCEP and 10% D2O to a final concentration of ∼300 μM. The chemical shifts of 1HN, 15 N, 13Cα, 13Cβ and 13CO cross-peaks were assigned using CBCA(CO)NH, HNCACB, HNCO and HN(CA)CO experiments and data were analysed using the program CCPNMR Analysis^40^. 93% of the amino acid side chain atoms were assigned. A similar procedure was followed to assign chemical shifts after formation of complexes between cPARP3 and DNA (oligonucleotides detailed in Supplementary Table 2).

### Nanogold labelling and electron microscopy

Full-length His-tagged cPARP3 was incubated for 30 min with intact or nicked nucleosomes. Ni-NTA-conjugated 5 nm gold (Nanoprobes, NY) was added at a molar ratio of 1:10 (cPARP3-Nucleosomes:Ni-gold). Samples were applied onto freshly glow-discharged carbon-coated grids and negatively stained with 2% (w/v) uranyl acetate. Electron micrographs were recorded at 30,000 nominal magnification on a camera GATAN model ULTRASCAN 1000 CCD using a HITACHI 7100 electron microscope operated at 100 kV.

### DT40 cells

To generate *PARP3*^*−/−*^ cells, genomic *PARP3* sequences were PCR amplified from DT40 genomic DNA (clone 18) to generate left and right arms for the targeting construct using the primers cPARP3-LA For/Rev for the left arm and cPARP3-RA (For/Rev) for the right arm. The PCR amplified products were subcloned into pCR2.1-TOPO vector (Invitrogen) and confirmed by Sanger sequencing. Fragments encoding the left arm (2.0 kb) and right arm (2.5 kb) were recovered from the above pCR2.1-TOPO construct using *Kpn*I/*Bam*HI and *Bam*HI/*Eco*R*I*, respectively, and subcloned into pCR2.1-TOPO vector. A *Bam*HI fragment encoding the neomycin (*Neo,* 2.4 kb) or hygromycin (*Hyg*, 3.4 kb) selection cassette was then inserted into the pCR2.1-TOPO construct at the *BamH*1 site separating the left and right arms, completing the *Neo*-selectable and *Hyg*-selectable *PARP3*-targeting constructs. To generate *PARP3*^*−/−*^ cells, 2 × 10^7^ wild-type DT40 cells (clone 18) were electroporated (Bio-Rad) with 30 μg of *Not*I-linearized *Neo*-targeting or *Hyg*-targeting construct and transfected clones selected for 8 days in the presence of medium containing 2.0 mg ml^−1^ G418 (Sigma-Aldrich) and/or 2.5 mg ml^−1^ hygromycin B (Sigma-Aldrich), as appropriate. To detect successful targeting of *PARP3* alleles, genomic DNA was isolated from drug-resistant clones, digested with *Xba*I *and Bam*HI, and subjected to Southern blot analysis using a 0.35 kb probe amplified from DT40 genomic DNA (clone 18) using the primers Probe-F & Probe-R (Supplementary Table 1). Following two consecutive rounds of *PARP3* gene targeting with constructs encoding resistance to G418 and hygromycin, respectively, we recovered one *PARP3*^*−/−*^ clone (denoted clone 36), which was characterized as indicated. For complementation experiments, the human PARP2 and PARP3 ORFs were amplified by PCR and cloned into the vector pCI-puro-N-Myc using the restriction sites *XhoI/NotI* and *XhoI/MluI* respectively. Point mutations in the WGR domain were generated by site-directed mutagenesis. *PARP3*^*−/−*^ cells (clone 36) were transfected by electroporation with pCI-puro-Myc-hPARP2, pCI-puro-Myc-hPARP3 or empty vector. Transfectants were selected in medium containing 0.5 μg ml^−1^ puromycin (Invitrogen) for 6–8 days. hPARP2 and hPARP3 expression were confirmed by western blot using anti-PARP2 (Active Motif cat. no. 39743; 1:1,000 dilution) or anti-PARP3 (rabbit #4699—a gift from Françoise Dantzer; 1:500 dilution).

### Alkaline comet assays

DT40 cells were treated with γ-rays (20 Gy), on ice. Where indicated, cells were subsequently incubated in drug-free complete media for the indicated repair periods. Cells were then suspended in pre-chilled PBS and mixed with an equal volume of 1.2% low-gelling-temperature agarose (Sigma, type VII) maintained at 42 °C. Cell suspension was immediately layered onto pre-chilled frosted glass slides (Fisher) pre-coated with 0.6% agarose and maintained in the dark at 4 °C until set, and for all further steps. Slides were immersed in pre-chilled lysis buffer (2.5 M NaCl, 10 mM Tris-HCl, 100 mM EDTA pH 8.0, 1% Triton X-100, 1% DMSO; pH10) for 1 h, washed with pre-chilled distilled water (2 × 10 min), and placed for 45 min in pre-chilled alkaline electrophoresis buffer (50 mM NaOH, 1 mM EDTA, 1% DMSO). Electrophoresis was then conducted at 1 V/cm for 25 min, followed by neutralization in 400 mM Tris-HCl pH7.0 for 1 h. Finally, DNA was stained with Sybr Green I (1:10,000 in PBS) for 30 min. Average tail moments from 50 cells/sample were measured using Comet Assay IV software (Perceptive Instruments, UK). Data are the average±1 s.e.m. of three independent experiments and were scored blind.

### Cell survival assay

Clonogenic survival was determined by colony formation assays. Briefly, DT40 cells were counted and plated in alpha-MEM medium containing 1.5% methylcellulose (w/v Sigma-Aldrich), 10% FBS, 1% chicken serum and 10 μM beta-mercaptoethanol. Cells were treated with γ-rays and after incubation for 9 days colonies that were visible by eye were counted. Survival was calculated by dividing the number of colonies in treated wells by those in untreated wells.

### UVA-laser micro-irradiation

ORFs encoding human PARP3-GFP and PARP3^CM^-GFP were generated by PCR amplification of the human PARP3 ORF present in pCD2E-PARP3 and pCD2E-PARP3^CM^ and subcloning into the *Eco*RI/*Sal*I sites of peGFP-N1. Point mutations in the WGR domain were generated by site-directed mutagenesis. Osteosarcoma U2-OS cells were transfected with GFP constructs 24 h before micro-irradiation, and incubated with 10 μg ml^−1^ Hoechst 34580 for 30 min before irradiation. Cells were micro-irradiated with a 405 nm UV-laser at a dose of 0.22 μJ μm^−2^ (ref. 42), and time-lapse images recorded at 10 s intervals for a total of 3 min per cell.

### Data availability

NMR assignment data have been deposited into the Biological Magnetic Resonance Data Bank with accession 26834. No other large data sets are associated with this work. All other data are available from the authors on request.

## Additional information

**How to cite this article:** Grundy, G. J. *et al.* PARP3 is a sensor of nicked nucleosomes and monoribosylates histone H2B^Glu2^. *Nat. Commun.* 7:12404 doi: 10.1038/ncomms12404 (2016).

## Supplementary Material

Supplementary Information Supplementary Figures 1-9 and Supplementary Tables 1-2.
