# Supplementary material for: PARP3 is a sensor of nicked nucleosomes and monoribosylates histone H2BGlu2
Source: Nat Commun. 2016 Aug 17;7:12404. doi: 10.1038/ncomms12404 (PMC4992063; doi:10.1038/ncomms12404)
Supplement: Supplementary Information — Supplementary Figures 1-9 and Supplementary Tables 1-2. [file ncomms12404-s1.pdf]

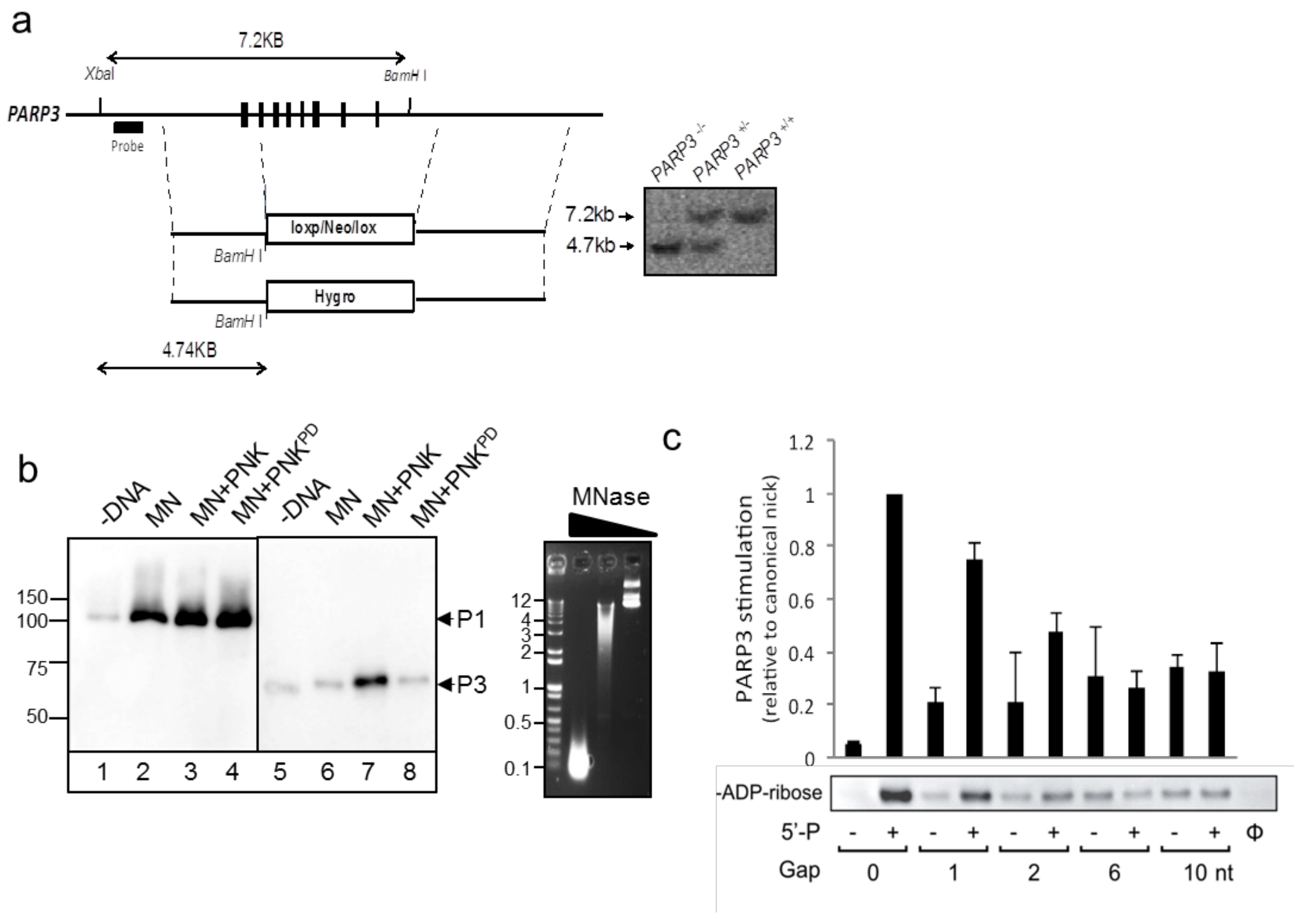


**Supplementary Figure 1**. (***a***), PARP3 was deleted in DT40 cells by targeted disruption, using the *neo* and *hygro* targeting constructs indicated. Southern blotting (using the probe indicated) was used to confirm disruption of the two *PARP3* alleles (*right)*. (***b***), PARP1 or PARP3 (50 nM) was incubated with biotin-NAD+ (12.5 μM) and MNase-digested plasmid for 30 min at room temperature and reaction products fractionated by SDS-PAGE and visualized as above. Where indicated, digested plasmid was pre-treated with wild type T4 PNK ("PNK") or "phosphatase-dead" T4 PNK ("$PNK^{PD}$") prior to use. *Right*, aliquots of plasmid DNA substrate treated with 0.15 U, 0.015 U, or 0.0015 U MNase (per 5 μg DNA) analysed by agarose gel electrophoresis and ethidium bromide staining. Plasmid digested with 0.015 U/5μg was employed for the PARP assays. Note that PNK 3'-posphatase activity is also required to activate the plasmid for PARP3 stimulation, indicating that non-canonical 3'-phosphate termini inhibit PARP3. **(*c*),** PARP3 (300 nM) was incubated for 20 min at room temp with biotin-NAD+ (12.5 μM) and a 32mer oligonucleotide duplex (200 nM) harbouring a single-strand gap of the indicated length. The presence (+) or absence (-) of phosphate at the 5'-terminus of the gap is indicated. All 5'-termini lacking phosphate were 5'-hydroxyl.

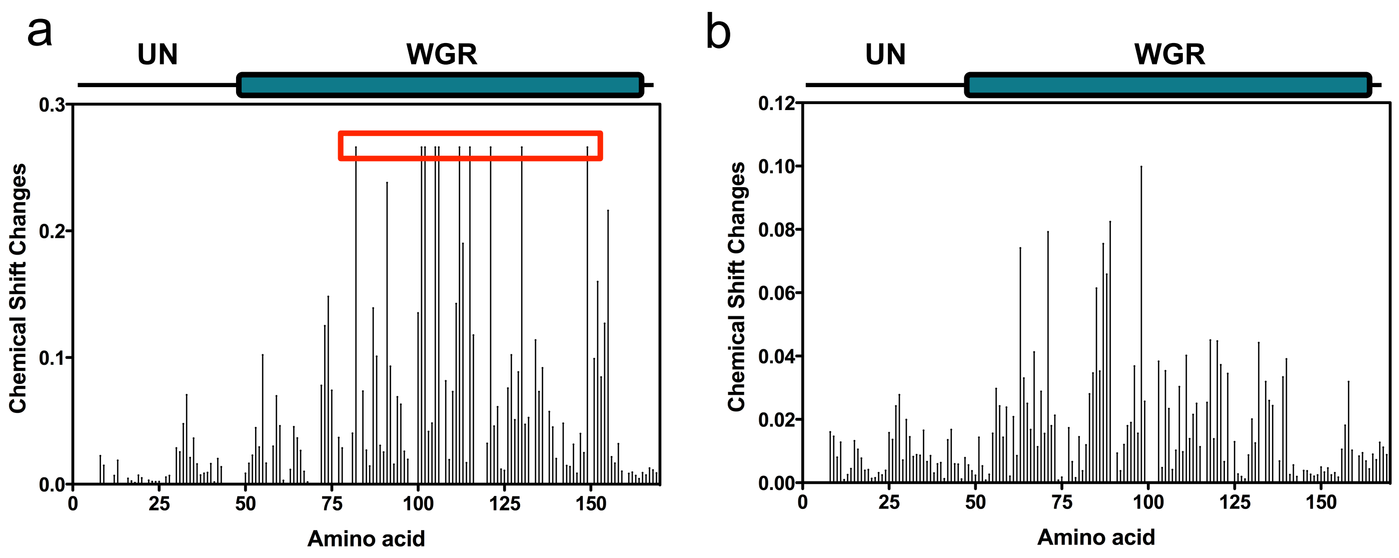


**Supplementary Figure 2. (A)** Plot of weighted chemical shift perturbations elicited by 5'-phosphorylated nicked oligonucleotide duplex, calculated using [($^1$H difference)2+(($^{15}$N difference)1/5))2]0.5 versus residue number for cPARP3 1-169. Residue signals that were not identified after titration are indicated with a red square. **(B)** Plot of weighted chemical shift perturbations elicited by 5'-phosphorilated 3'-overhang oligonucleotide duplex, calculated using [($^1$H difference)2+(($^{15}$N difference)1/5))2]0.5 versus residue number for cPARP3 1-169.

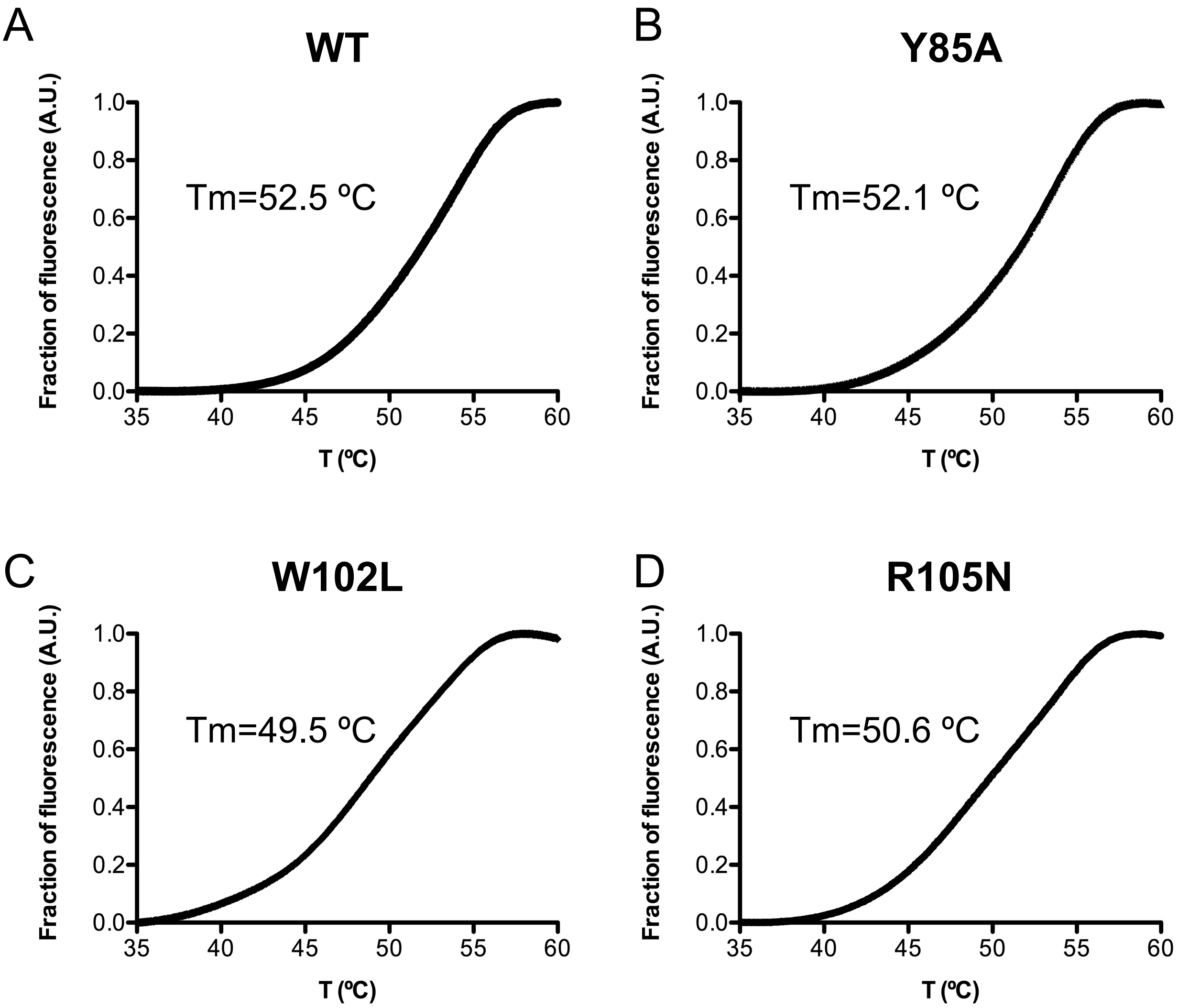


**Supplementary Figure 3.** Thermostability of (**a**) wild type cPARP3, (**b**) cPARP3$^{Y85A}$, (**c**) cPARP3$^{W102L}$, and **(d)** cPARP3$^{R104N}$. Solutions containing protein at 6 μM concentration were screened in triplicate in 25 mM HEPES pH 7.4 and 125 mM NaCl. A thermal ramping procedure (1 ºC/min over a range of 25 ºC to 80 ºC) was employed.

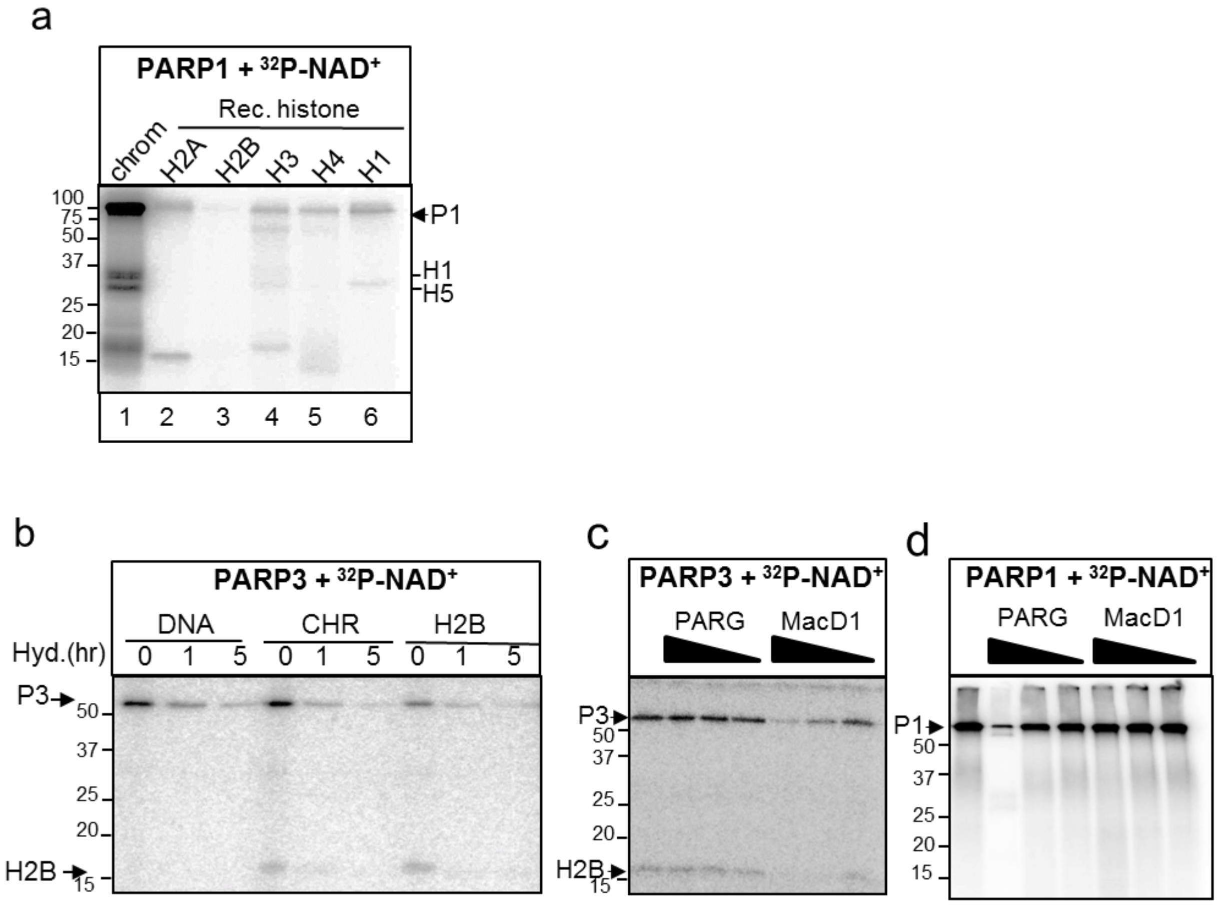


**Supplementary Figure 4.** (**a**), 1 μg of the indicated recombinant histone or chicken chromatin was incubated with 100 nM PARP1 in the presence of 300 nM $^{32}$P-NAD$^{+}$ and oligonucleotide harbouring a DSB and the reaction products separated by 15% SDS-PAGE and detected by autoradiography. **(b),** The products of PARP3 (100 nM) ribosylation reactions employing 1 μM nicked DNA, 300 nM $^{32}$P-NAD$^{+}$, and either 100 ng ml$^{-1}$ chromatin or recombinant H2B were subject to neutral hydroxylamine treatment (1 M) for the indicated times, fractionated by SDS-PAGE, and detected as above. **(c),** The products of PARP3 (100 nM) ribosylation reactions employing 1 μM nicked DNA, 1 μM $^{32}$P-NAD$^{+}$, and either 1 μg chromatin or recombinant H2B were stopped with 1 mM 3-aminobenzamide, incubated for 30 min at room temp with PARG (100 pg, 20 pg or 4 pg) or His-MACROD1 (280 nM, 44 nM or 9 nM) and analysed as above. (**d**), The products of PARP1 ribosylation actions were conducted and analysed as in panel c.

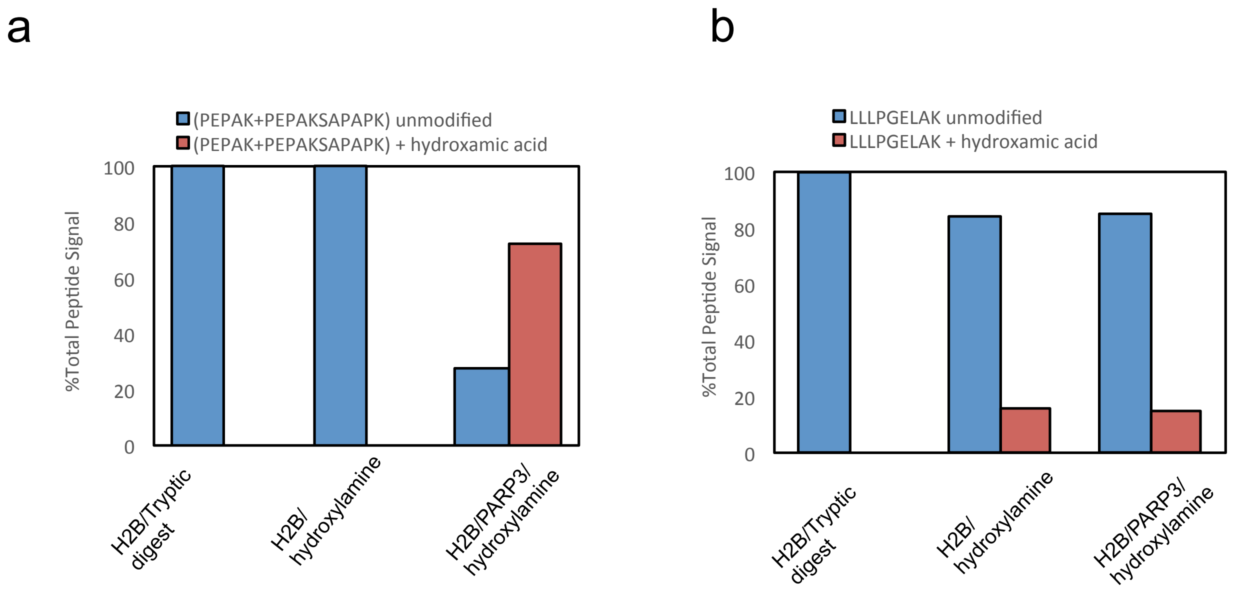


**Supplementary Figure 5.** Sites of PARP3 ribosylation in recombinant H2B. **(a & b),** Quantification of the indicated unmodified or hydroxamic acid (+15.0109 Da) modified peptides identified in H2B samples following either tryptic digest, tryptic digest/hydroxylamine treatment, or PARP3 treatment/tryptic digest/boronate affinity chromatography (to enrich ADP-ribosylated peptides)/hydroxylamine treatment. A tryptic digest/boronate affinity chromatography/hydroxylamine control sample lacking PARP3 pre-treatment was conducted in parallel but is omitted from the quantification because, as expected, insufficient H2B peptide was recovered in the affinity enrichment step for accurate quantification.

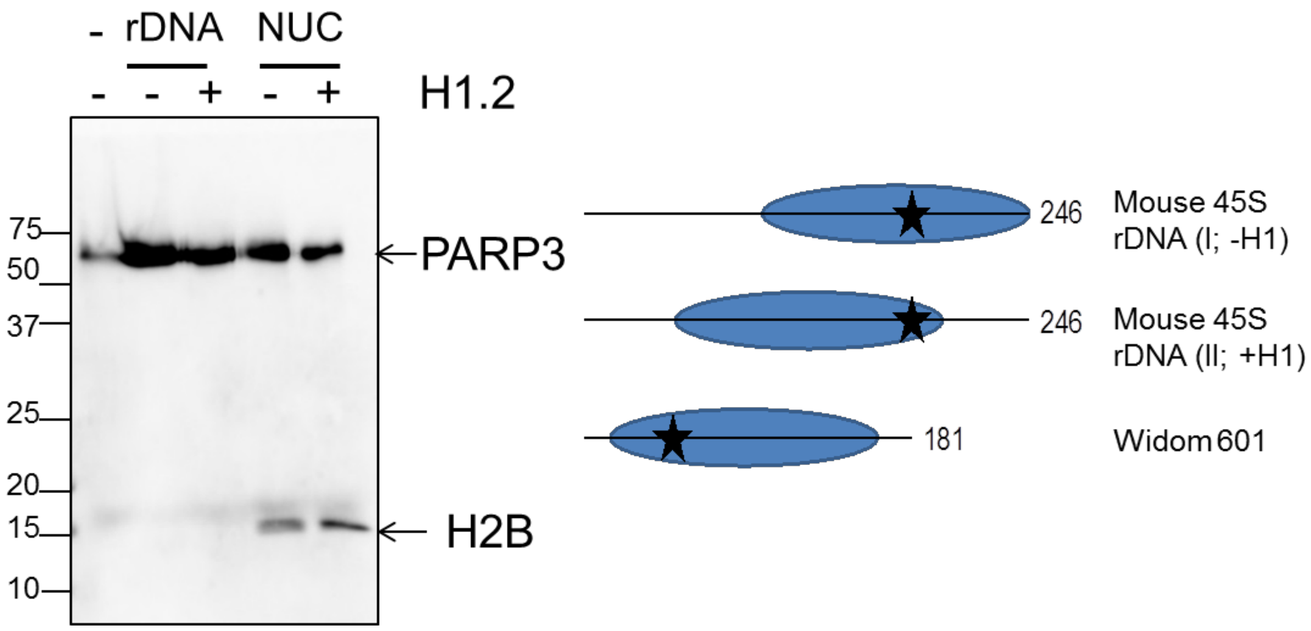


**Supplementary Figure 6.** *Right*, cartoon showing two major positions of nucleosomes adopt on a 246-bp mouse 45S rDNA fragment Type-I; in which the site-specific nick induced by Nt.BsmAI is located centrally within the nucleosome or Type-II; in which the nick is located at he exit of the nucleosome. Addition of Histone H1 favours the Type II conformation. *Left*, PARP3 (100 nM) was incubated with biotin-NAD+ (12.5 μM) and the 246-bp rDNA (100 nM) present either as naked DNA or as Type I or Type II nucleosomes as indicated for 30 min. at room temperature. Reaction products were separated by 15% SDS-PAGE and biotinylated protein detected by western blot using streptavidin-HRP.

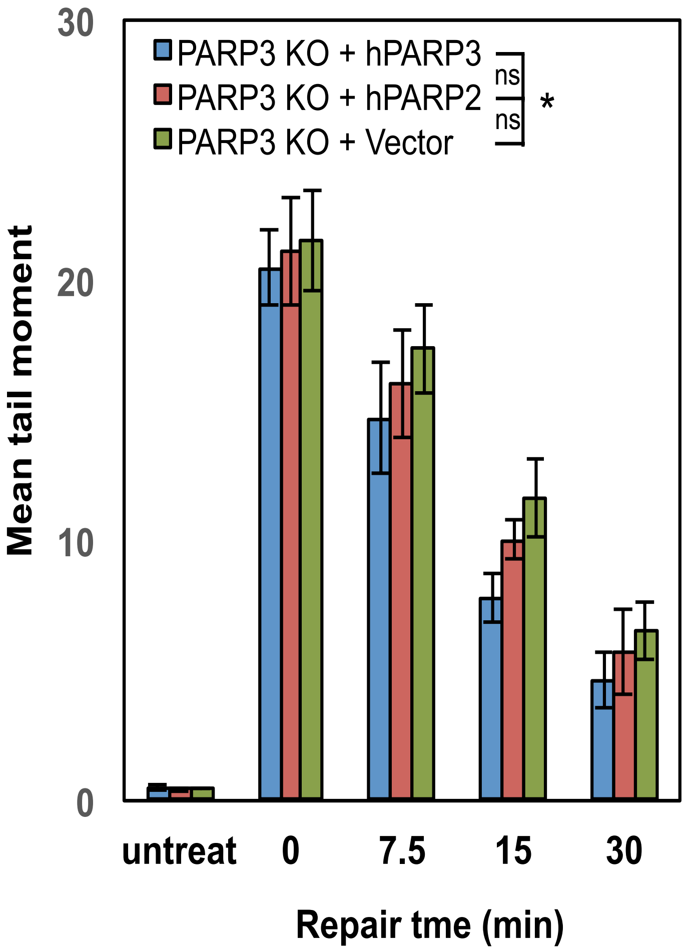


**Supplementary Figure 7**. *PARP3*$^{-/-}$ DT40 cells (denoted "KO") stably transfected with empty vector or vector encoding human PARP3 (hPARP3) or human PARP2 (hPARP2) were left untreated or treated on ice with γ-rays (20 Gy) and incubated for the indicated times to allow repair. DNA strand breaks were quantified (average tail moment) by alkaline comet assays from >50 cells per sample and data are the mean (+/-S.E.M.) of three independent experiments. Statistically insignificant (ns) or significant (*p<0.05) differences are indicated (ANOVA)

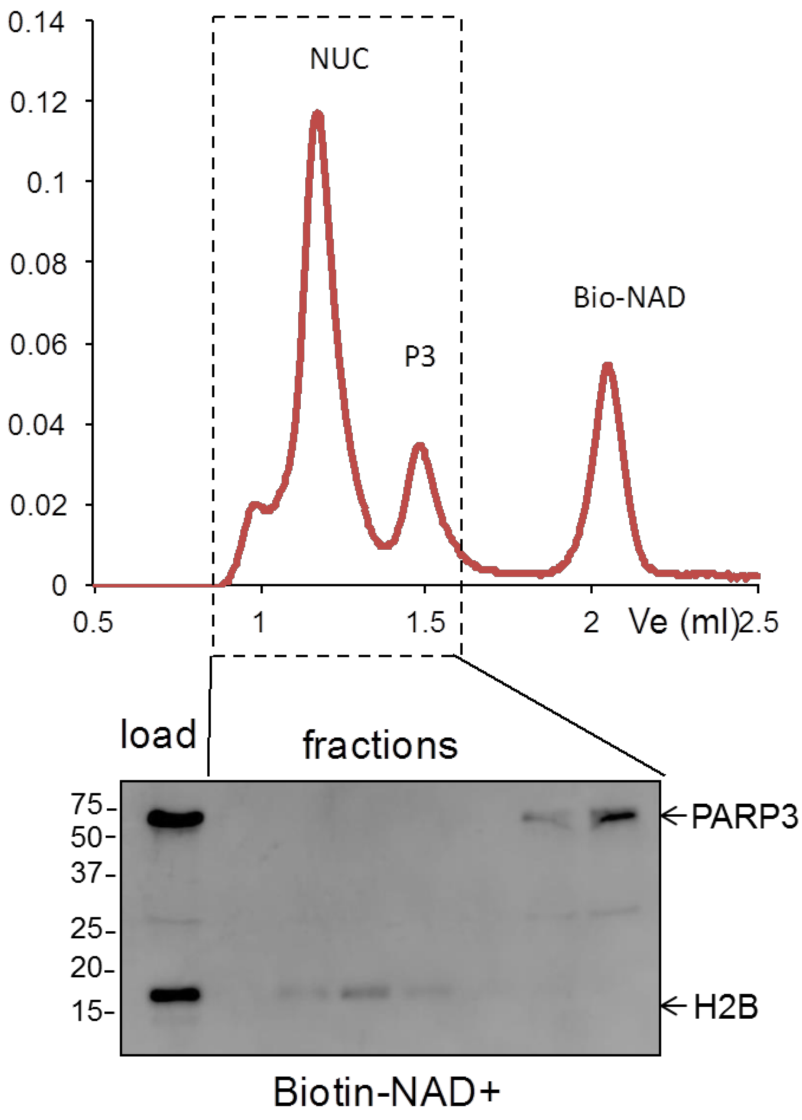


**Supplementary Figure 8.** PARP3 ADP-ribosylated nucleosomes were separated by gel filtration and the fractions probed for biotinylated PARP3 and H2B by western blot. 300 nM nucleosomes assembled on nicked 601.2 DNA were ribosylated with 300 nM PARP3 and 12.5 μM Biotin-NAD+ for 30min at room temperature. 50 μl was immediately injected onto Superdex 200 (10/3.2 column, GE Healthcare) using 10 mM Tris pH7.5, 0.2 M NaCl running buffer at 50 μl/ml (SMART system, GE Healthcare). 100 μl fractions were analysed by 15% SDS-PAGE/western blot using streptavidin-HRP. Note that ADP-ribosylated H2B was detected in the nucleosome fractions.

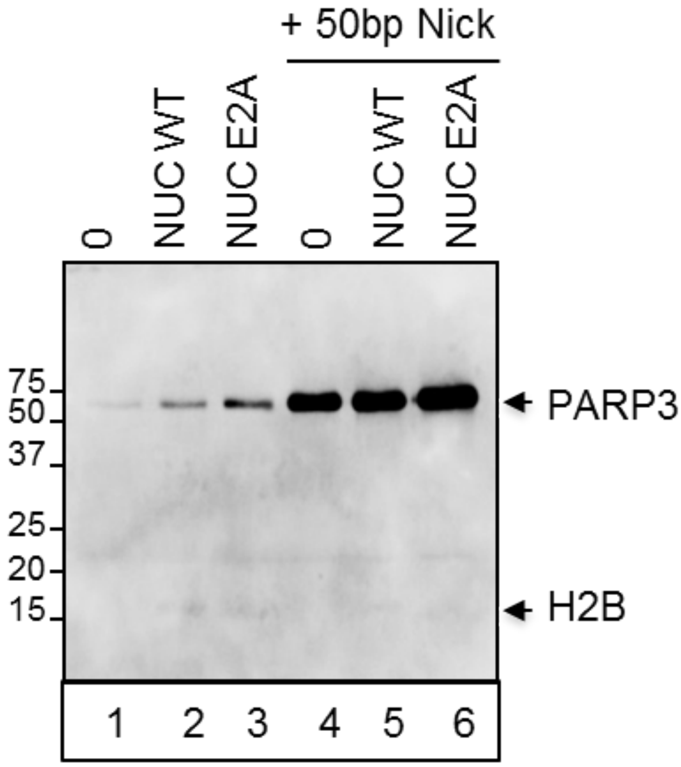


**Supplementary Figure 9**. PARP3 ribosylates H2B *in cis*. Reactions containing wild type or mutant intact nucleosomes (100 nM) were incubated with 100 nM PARP3 and 12.5 μM biotin-NAD+ in the absence (lanes 1-3) or presence (lanes 4-6) of 50-bp naked nicked oligonucleotide duplex (100 nM).

## Supplementary Tables

**Supplementary Table 1:** Oligonucleotide sequences for gene cloning and mutagenesis. START codons are in bold. Mutated residues are in red.

| Name | Forward Primer (denoted Fw or F) | Reverse Primer (denoted Rv or R) |
|---|---|---|
| cPARP3 pNIC28 | TACTTCCAATCC**ATG**GCCCCGAAACGTC | pNIC28-Rv; TATCCACCTTTACTGttaGAAGTGCAGTTGAACCAGG pNIC28-169Rv; TATCCACCTTTACTGTTAATCCACACGCAGAGCGACTTC |
| cPARP3 Y85A | AAATTCGCCATCATCCAACTGATCGAAC | GATGATGGCGAATTTGTTGTTGTTCGCTG |
| cPARP3 W102L | CCGTCTGGGCCGCGTTGGTG | GCCCAGACGGTTCCACGTCGAG |
| cPARP3 R104N | GGCAACGTTGGTGAAGTCGGC | CCAACGTTGCCCCAACGGTTCCAC |
| hPARP3 Y83A | CAAGTTCGCCATCATCCAGCTGCTCCAAGAC | GATGATGGCGAACTTGTTGTTGTTGTTCTCGATG |
| hPARP3 W101L | CCGCCTGGGCCGTGTGGGAGAGGTC | GGCCCAGGCGGTTCCAGCAGGTGAAGAAG |
| hPARP3 R103L | GGGCAATGTGGGAGAGGTCGGCCAG | CCCACATTGCCCCAGCGGTTCCAGCAG |
| H2B | CTCAACGC**ATG**CCTGAGCCAGCGAAATCC | ATGCTACTCGAGTTACTTGGAGCTGGTGTACTTGGTAAC |
| pET28a-H2B-E2A | GAAGGAGATATACC**ATG**CCTGCACCAGCTAAGTCAGCTCCTG | CAGGAGCTGACTTAGCTGGTGCAGGCATGGTAT ATCTCCTTC |
| cPARP3-LA | CTCAGCAGCTCTTACAGTGAACTC | TGAGCTGGATGATGTAGAACTTGT |
| cPARP3-RA | CCAATAAACCCACTGTGCTTC | GGTGTTATGGTGTTAGTGGTGTTATC |
| Probe F & R | CAGGCGAATCTCTCTCCAAC | CCAGCCCAAAAGAGTATCCA |

**Supplementary Table 2: Oligonucleotide sequences for DNA substrates**

| Gap*n* top 5’ *n* Gap top 3’ / Gap bottom | Sequence 5’-3’ |
|---|---|
| Gap 3’top | GGCTGTGC |
| Gap 3’top 5’P | **P**-GGCTGTGC |
| Gap*0* 5’top | CCAACGACTCCAAGCATCGCTCAC |
| Gap*1* 5’top | CCAACGACTCCAAGCATCGCTCA |
| Gap*2* 5’top | CCAACGACTCCAAGCATCGCTC |
| Gap*6* 5’top | CCAACGACTCCAAGCATC |
| Gap*10* 5’top | CCAACGACTCCAAG |
| Gap bottom | GCACAGCCGTGAGCGATGCTTGGAGTCGTTGG |
| 25N1 25N2 / 50 comp | Sequence 5’-3’ |
| 25N1 | GGGAGAACTGATAAGACCGACAGGT |
| 25N1P | GGGAGAACTGATAAGACCGACAGGT-**P** |
| 25N2 | AATTCTTCTCTTTCCAGGGCTATGT |
| 25N2P | **P**-AATTCTTCTCTTTCCAGGGCTATGT |
| 50comp | ACATAGCCCTGGAAAGAGAAGAATTACCTGTCGG TCTTATCAGTTCTCCC |
| Gap*0* NMR top 5’ Gap*0* NMR top 3’ / Gap*0* NMR bottom | Sequence 5’-3’ |
| Gap0 NMR bottom | GCACAGCCGTGAGCGATGC |
| Gap0 NMR 5’top | GCATCGCTCA |
| Gap0 NMR 3’top | **P**- CGGCTGTGC |
